# Compressive Strength and Chloride Ion Penetration Resistance of GGBFS-Based Alkali-Activated Composites Containing Ferronickel Slag Aggregates

**DOI:** 10.3390/ma17194922

**Published:** 2024-10-09

**Authors:** Jae-In Lee, Chae-Young Kim, Joo-Ho Yoon, Se-Jin Choi

**Affiliations:** Department of Architectural Engineering, Wonkwang University, 460 Iksan-daero, Iksan 54538, Republic of Korea; jaein0482@daum.net (J.-I.L.); cykim043@naver.com (C.-Y.K.); yoonjh0091@naver.com (J.-H.Y.)

**Keywords:** ferronickel slag aggregate, alkali-activated composites, mortar flow, compressive strength, chloride ion penetrability

## Abstract

Various studies have reported the use of alkali-activated composites to enable sustainable development in the construction industry as these composites eliminate the need for cement. However, few studies have used ferronickel slag aggregates (FSAs) as an aggregate material for alkali-activated composites. Alkali-activated composites are environmentally friendly and sustainable construction materials that can reduce carbon dioxide emissions from cement production, which accounts for 7% of global carbon emissions. In the construction industry, various research was conducted to improve the performance of alkali-activated composites, such as changing the binder, alkali activator, or aggregate. However, research on the application of ferronickel slag aggregate as an aggregate in alkali-activated composites is still insufficient. In addition, the effect of ferronickel slag aggregate on the performance of alkali-activated composites when using calcium-based or sodium-based alkali activators has not been reported yet. Thus, this study prepared ground granulated blast-furnace slag-based alkali-activated composites with 0, 10, 20, and 30% FSA as natural fine aggregate substitutes. Then, the fluidity, micro-hydration heat, compressive strength properties, and resistance to chloride ion penetration of the alkali-activated composite were evaluated. The test results showed that the maximum temperature of the CF10, CF20, and CF30 samples with FSA was 35.4–36.4 °C, which is 3.8–6.7% higher than that of the CF00 sample. The 7 d compressive strength of the sample prepared with CaO was higher than that of the sample prepared with Na_2_SiO_3_. Nevertheless, the 28 d compressive strength of the NF20 sample with Na_2_SiO_3_ and 20% FSA was the highest, with a value of approximately 55.0 MPa. After 7 d, the total charge passing through the sample with Na_2_SiO_3_ was approximately 1.79–2.24 times higher than that of the sample with CaO. Moreover, the total charge decreased with increasing FSA content.

## 1. Introduction

Recently, numerous researchers in the construction industry have investigated alkali-activated composites to reduce the environmental pollution caused by cement production as these composites do not require cement [1,2,3]. A large amount of carbon dioxide is emitted during the cement production process, which accelerates global warming and causes environmental destruction. In addition, the mining of limestone, which is used as the main raw material for cement, causes destruction of the ecosystem and depletion of natural resources. The binders used in alkali-activated composites are primarily aluminosilicate materials, including ground granulated blast-furnace slag (GGBFS) and fly ash, which are rich in SiO_2_ and Al_2_O_3_ [4,5,6,7].

Alkaline activators are used to initiate the polymerization of binders that do not have intrinsic hydraulic properties [8]. Some studies have reported that the performance of alkali-activated composites varies depending on the type and amount of the alkaline activator used [9,10,11].

Prochon et al. [9] investigated the mechanical properties of geopolymer mortars using different alkali activators, such as CaO, NaOH, and Na_2_SiO_3_. The study reported that samples containing a mixture of NaOH and Na_2_SiO_3_ exhibited the highest compressive strength. In contrast, the compressive strength of samples containing only a single alkali activator presented a delayed development.

Singh et al. [10] examined the microstructures of geopolymer mortars using 2M and 12M alkali activators. The study result indicated that the sample with a 2M concentration showed a dense matrix, whereas the sample using a 12M concentration had voids containing unreacted activators due to excessive alkaline activators. Moreover, the compressive strength of the sample with the 12M concentration was lower than that of the sample with the 2M concentration. Rocha et al. [11] reviewed the properties of metakaolin-based geopolymer mortars fabricated using various alkaline activators. In particular, a matrix with many voids was observed in samples with sodium silicate, and a matrix with high density and few voids was observed in samples with potassium silicate.

Furthermore, conserving natural resources is becoming crucial for sustainable development of the construction industry [12]. For aggregates with limited natural resources, depletion increases as the demand for concrete in large-scale infrastructure production increases [13]. Recently, ferronickel slag aggregate (FSA) generated in the steel industry has been used as a substitute for natural aggregates in concrete to conserve natural aggregates and promote the use of steel industry byproducts. In particular, several studies have indicated that cement composites containing FSA have excellent chloride ion penetration and sulfate resistance [14,15,16].

Lee et al. [14] examined the effect of the ferronickel slag fine aggregate replacement rate on the properties of cement mortar. The results indicated that using ferronickel slag fine aggregate improved the fluidity of cement mortar. Moreover, the sample with 50% ferronickel slag fine aggregate as a substitute for natural fine aggregate showed the highest resistance to chloride ion penetration. Saha et al. [15] analyzed the mechanical properties of structural concrete using ferronickel slag fine aggregate. The study revealed that adding 50% ferronickel slag fine aggregate to concrete maximized its compressive strength and improved its workability. Bao et al. [16] investigated the freeze–thaw resistance of recycled aggregate concrete by including ferronickel slag fine aggregate. As a result, concrete containing 40% ferronickel slag fine aggregate had excellent freeze–thaw resistance.

However, few studies have used FSA as an aggregate material in alkali-activated composites, and most of them have primarily used sodium-based activators; however, none have employed calcium-based activators. Therefore, in this study, calcium and sodium-based alkali-activated composites containing 0, 10, 20, and 30% FSA were prepared, and the effect of FSA on their performance was investigated. Subsequently, the fluidity, micro-hydration heat, compressive strength properties, and chloride ion penetration resistance of the alkali-activated composite were evaluated.

## 2. Materials and Methods

### 2.1. Materials

Ground granulated blast-furnace slag (GGBFS) (Daehan slag, Gwangyang, Republic of Korea) and fly ash (Dangjin Thermal Power Plant, Dangjin, Republic of Korea) were used. Alkali activators, including CaO, Na_2_SiO_3_, CaCl_2_, Ca(HCOO)_2_, and NaOH (Daejung, Republic of Korea), were in granular form as described previously [17]. Natural sand (NSA) with a specific gravity of 2.60 g/cm^3^ and a fineness modulus (FM) of 2.45 and FSA (POSCO, Pohang-si, Republic of Korea) with a specific gravity of 3.04 g/cm^3^ and FM 3.64 were used as fine aggregates.

**Figure 1:**
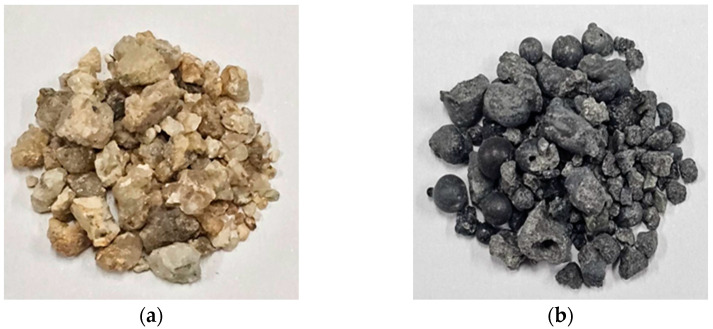
Shapes of aggregates. (**a**) NSA and (**b**) FSA.

**Figure 2:**
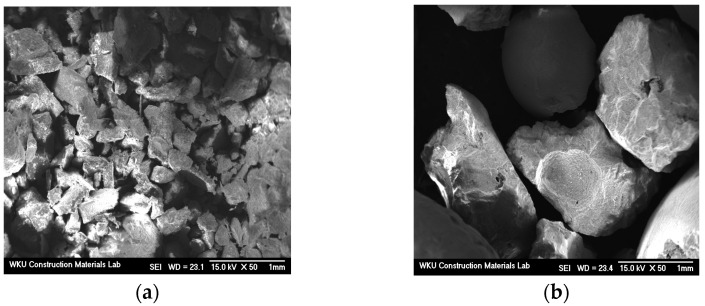
SEM images of aggregates. (**a**) NSA and (**b**) FSA.

Table 1 and Table 2 list the chemical composition of the binder and the physical properties of the fine aggregate used in this study. Table 3 lists the chemical composition of FSA measured by energy-dispersive X-ray spectroscopy (EDS). Notably, its composition comprises Si, Mg, Al, and Fe. Figure 1 and Figure 2 show the shapes and SEM images of NSA and FSA, respectively.

**Figure 3:**
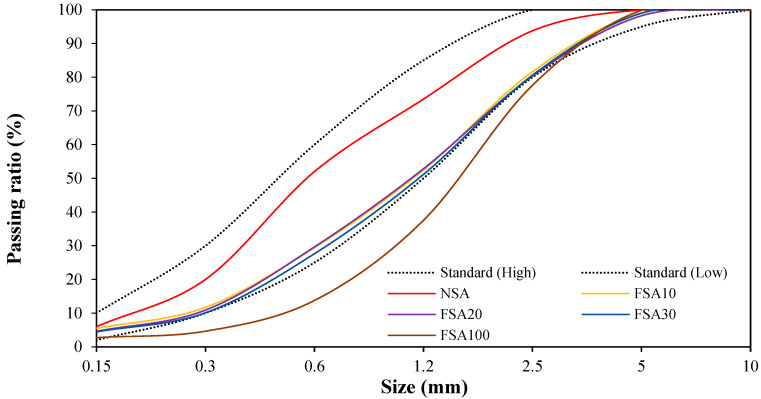
Particle size distribution of fine aggregates.

Figure 3 shows the particle size distribution curves of the fine aggregates. The figure shows that FSA100, which solely used FSA, exceeds the standard particle size distribution curve range. However, the mixed fine aggregates (FSA10, FSA20, and FSA30), substituting 10, 20, and 30% FSA for NSA, conform to the standard particle size distribution curve range.

### 2.2. Mix Proportions and Specimen Preparation

Table 4 lists the mix proportions of the GGBFS–based alkali-activated composites with NSA for a water/binder ratio (W/B) of 40%. FSA was used as a substitute for NSA at 0, 10, 20, and 30% (by volume). The amount of alkaline activator was selected in a preliminary experiment. CaCl_2_, Ca (HCOO)_2_, and NaOH were fixed at 5%, 5%, and 2.5%, respectively. Subsequently, the samples were divided into those with 2.5% CaO (CF00, CF10, CF20, and CF30) and those with 2.5% Na_2_SiO_3_ (NF00, NF10, NF20, and NF30). Depending on the amount of FSA, the samples were labeled as F00 (FSA 0%), F10 (FSA 10%), F20 (FSA 20%), and F30 (FSA 30%). To evaluate the compressive and tensile strength of the GGBFS–based alkali-activated composite using FSA, a cubic specimen of 50 × 50 × 50 mm and a cylindrical specimen of Ø50 × 100 mm were prepared. Moreover, a Ø100 × 50 mm cylindrical test specimen was prepared to evaluate resistance to chloride ion penetration.

### 2.3. Experimental Procedures and Equipment

Micro-structural analyses were performed using scanning electron microscopy (SEM) and energy-dispersive X-ray spectroscopy (EDS). After 28 d, a thermogravimetric analysis (TGA) was performed on various samples at a maximum temperature of 900 °C for approximately 90 min. After the samples were prepared, they were cured in a steam-curing chamber at 40 °C and 100% relative humidity to the desired age.

**Figure 4:**
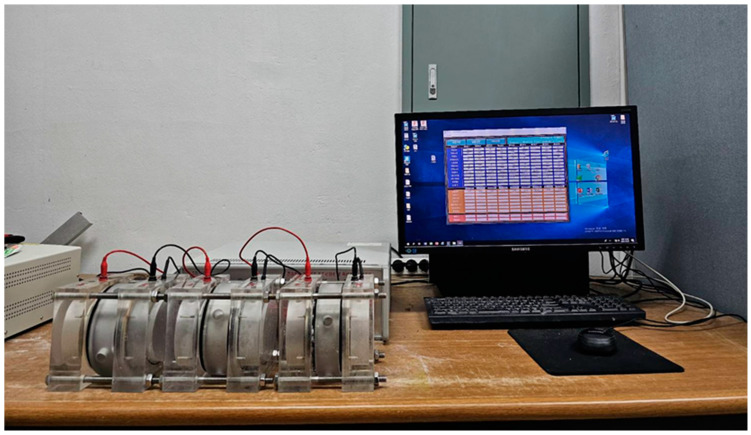
Chloride ion permeability test device.

Mortar flow and compressive strength were measured in accordance with KS L 5105 [18]. Micro-hydration heat was measured in accordance with ASTM C 1753 [19] using a semi-adiabatic calorimeter (F-Cal8000, Calmetrix, Boston, MA, United States). Splitting tensile strength was determined according to KS F 2423 [20], and chloride ion penetration resistance was measured according to ASTM C 1202 [21] (Figure 4). Microstructure was analyzed by SEM (AIS1800C, SERON, Seoul, Republic of Korea) and EDS (OXFORD INSTRUMENTS, High Wycombe, UK), and TGA was performed using a Thermal Analyzer (TGA N-1000, Scinco, Seoul, Republic of Korea).

## 3. Results

### 3.1. Mortar Flow

**Figure 5:**
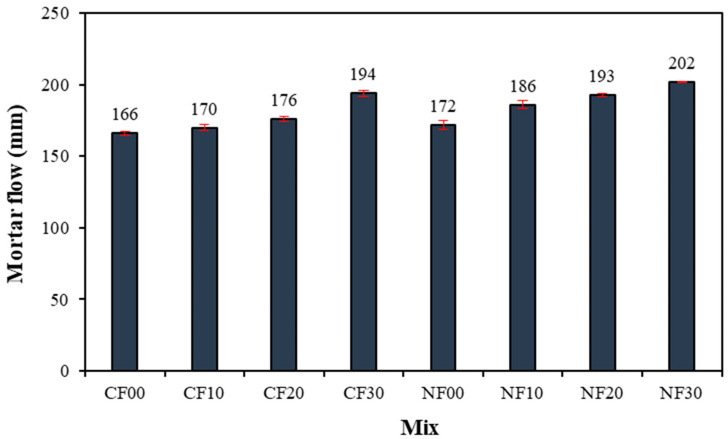
Mortar flow.

Figure 5 shows the flow change in GGBFS–based alkali-activated mortar using FSA. In particular, the flow of the CF00 sample with 2.5% CaO and 0% FSA was the lowest at approximately 166 mm. Moreover, as the amount of FSA increased, the mortar flow increased. The flow of the alkali-activated sample using FSA was approximately 2.4–16.8% higher than that in the CF00 sample. Note that even for the samples prepared with 2.5% Na_2_SiO_3_, the mortar flow increased with increasing FSA content. As shown in Table 2, the absorption rate of FSA is approximately 0.6%, which is lower than that of NSA (1.0%); therefore, the remaining excess water is assumed to increase mortar flow [22].

In addition, at a constant amount of FSA, the mortar flow of the sample with Na_2_SiO_3_ as the activator was larger than that of the sample with CaO.

### 3.2. Heat of Micro-Hydration

**Figure 6:**
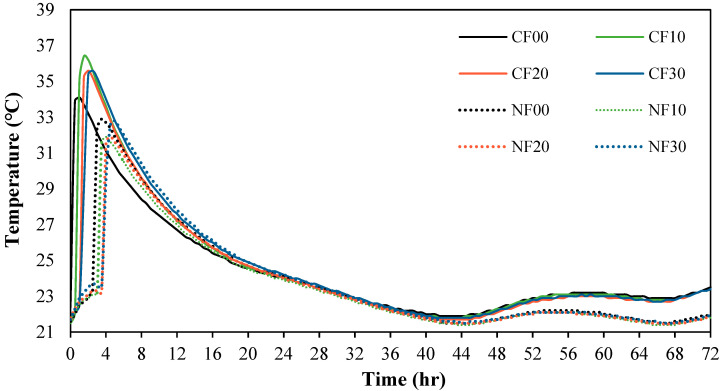
Heat of micro-hydration.

Figure 6 shows the change in the heat of micro-hydration of the alkali-activated mortar samples using FSA. The maximum temperature of the CF00 sample with CaO and 0% FSA was approximately 34.1 °C after 60 min.

Moreover, the maximum temperature of the CF10, CF20, and CF30 samples with FSA was between 35.4 and 36.4 °C, which was approximately 3.8–6.7% higher than that of the CF00 sample. For samples with Na_2_SiO_3_, the maximum temperature was comparable at temperatures between 31.9 and 32.8 °C regardless of the amount of FSA. Furthermore, the time to reach the maximum temperature tended to be slightly delayed for both the CaO and Na_2_SiO_3_ samples as the FSA content increased. This result might be because the absorption rate of FSA is relatively lower than that of NSA, increasing the free water [23].

In particular, the heat of micro-hydration was greater for the sample with CaO than for the sample with Na_2_SiO_3_. This is because the elution of Ca^2+^ ions accelerated the polymerization reaction [24,25].

### 3.3. Compressive Strength

**Figure 7:**
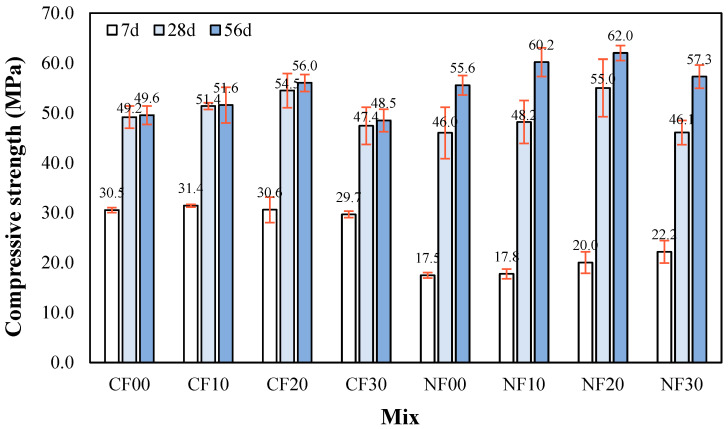
Compressive strength.

Figure 7 shows the change in compressive strength of the alkali-activated composite prepared with FSA with respect to age. The 7 d compressive strength of the sample using CaO was relatively higher than that of the sample with Na_2_SiO_3_ and ranged from 29.7 to 31.4 MPa regardless of the amount of FSA. The 7 d compressive strength of the Na_2_SiO_3_ sample was the highest at approximately 22.2 MPa for the NF30 sample with 30% FSA.

The reason that the 7 d compressive strength of the CaO sample was higher than that of the Na_2_SiO_3_ sample might be due to the polymerization reaction caused by Ca ion elution, which is similar to the change in micro-hydration heat [24].

For the samples with CaO, the compressive strength of the CF20 sample after 28 d was approximately 54.5 MPa, which was higher than that of the other samples with CaO. The 28 d compressive strength of the CF30 sample using 30% FSA was approximately 47.4 MPa, which was approximately 13.0% lower than that of the CF20 sample.

The 28 d compressive strength of the NF20 sample with Na_2_SiO_3_ and 20% FSA was highest at approximately 55.0 MPa. In addition, the 28 d compressive strength of the NF30 sample with 30% FSA was approximately 46.1 MPa, which was approximately 16.1% lower than that of the NF20 sample.

The 28 d compressive strength of the sample with Na_2_SiO_3_ showed a higher increasing rate than that of the sample with CaO. This is because the internal structure becomes denser with age due to the formation of N–A–S–H gel by the reaction of Ca, Al, and Si. [26,27,28,29,30]. Additionally, the relatively low compressive strength of the sample with 30% FSA was owing to a reduced packing effect due to the large particles of FSA. This trend is similar to that reported in previous studies [31,32] where the compressive strength decreased when the FSA content exceeded 25%. Moreover, this trend continued after 56 d where compressive strengths of the CF20 and NF20 samples with 20% FSA were relatively high at approximately 56.0 and 62.0 MPa, respectively. In addition, the 56 d compressive strength of the sample with Na_2_SiO_3_ was approximately 10.7–18.1% higher than that of the sample with CaO at the same FSA content.

### 3.4. Split-Tensile Strength

**Figure 8:**
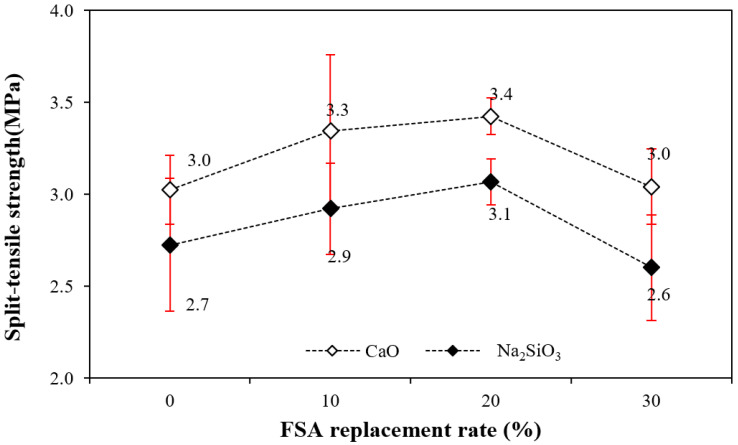
Split-tensile strength.

Figure 8 shows the change in the 28 d tensile strength of the alkali-activated composite as a function of the amount of FSA. The figure shows that the tensile strength of the sample with CaO was approximately 9.6–15.3% higher than that of the sample with Na_2_SiO_3_ regardless of the amount of FSA.

Similar to the compressive strength trend, the tensile strength increased when the amount of FSA increased to 20%, and the tensile strength of the sample with 30% FSA decreased. That is, the tensile strength of the sample with 20% FSA was 3.4 (sample with CaO) and 3.1 MPa (sample with Na_2_SiO_3_), which was approximately 13.3–14.8% higher than that of the sample with 0% FSA.

However, the tensile strength of the sample with 30% FSA was similar to that of the sample without FSA.

### 3.5. Chloride Ion Penetrability

**Figure 9:**
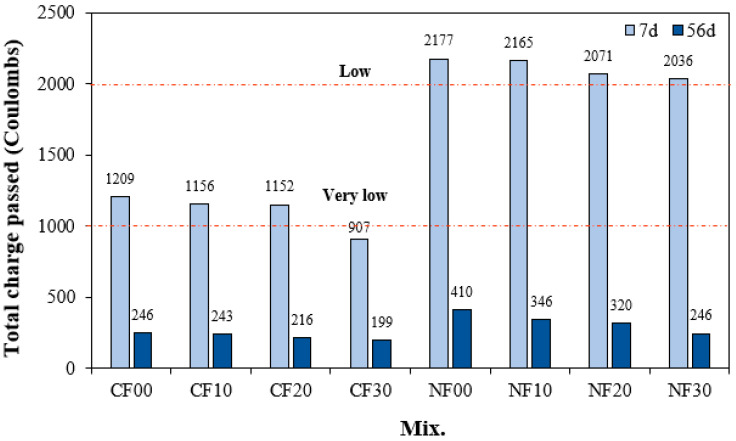
Chloride ion penetrability.

Figure 9 shows the change in chloride ion permeability of the alkali-activated composites with FSA. After 7 d, the total charge passing through the sample with Na_2_SiO_3_ was approximately 1.79 to 2.24 times higher than that of the sample with CaO. In particular, the total charge passing through the NF00 sample without FSA was the largest at approximately 2177 C. Moreover, the total charge decreased as the amount of FSA increased. The chloride ion penetration resistance of the NF30 sample with 30% FSA was approximately 6.4% higher than that of the NF00 sample without FSA.

The total charge passing through the sample with CaO was between 907 and 1209 C, showing better chloride ion penetration resistance than the sample using Na_2_SiO_3_. In particular, the total charge of the CF30 sample with 30% FSA is approximately 907 C, which corresponds to a “very low” value according to ASTM C 1202.

Even for the CaO samples, the total charge decreased as the FSA content increased. This result might be because as the FSA content increases, the internal density of the alkali-activated composites increases, preventing the penetration of chloride ions [33,34,35,36], and the C–S–H gel produced by the reaction of FSA makes the ITZ region dense [16,37].

In addition, the total charge of the sample with CaO was lower than that of the sample with Na_2_SiO_3_ because the Ca content was high, and a relatively large amount of hydrate was generated [38].

After 56 d, the total charge values of all samples decreased significantly. That is, the total charge of the sample with CaO was between 199 and 246 C, and the total charge of the sample with Na_2_SiO_3_ was between 246 and 410 C after 56 d, indicating excellent resistance to chloride ion penetration. Moreover, the total charge value of the 56 d sample also decreased with increasing FSA content.

### 3.6. Microstructural Analysis

**Figure 10:**
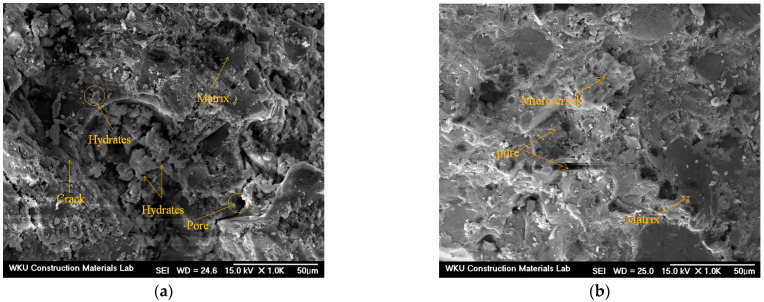

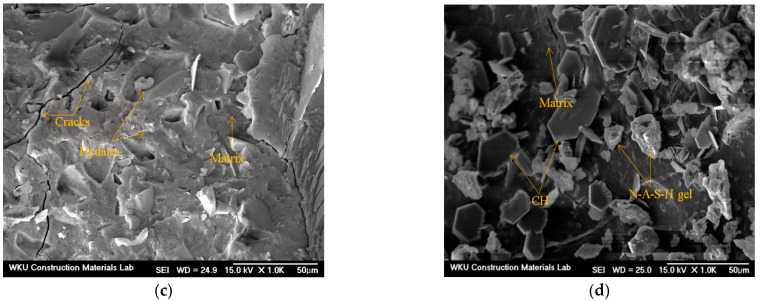
SEM images of 28 d samples. (**a**) CF00, (**b**) CF20, (**c**) NF00, and (**d**) NF20.

Figure 10 shows the SEM images of samples after 28 d. Notably, microcracks and hydrates can be observed in the CF00 sample with CaO and 0% FSA. Furthermore, the CF20 sample with 20% FSA presents fewer cracks and a denser surface than the CF00 sample. Because of this microstructure, the compressive and tensile strengths of the CF20 sample were higher than those of the CF00 sample. For samples with Na_2_SiO_3_, cracks are observed in the NF00 sample without FSA, and N–A–S–H gel hydrate with a dense surface is observed in the NF20 sample with 20% FSA.

**Figure 11:**
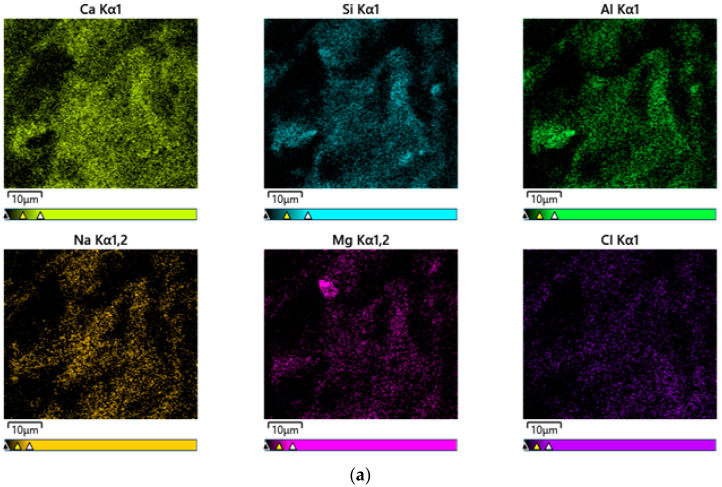

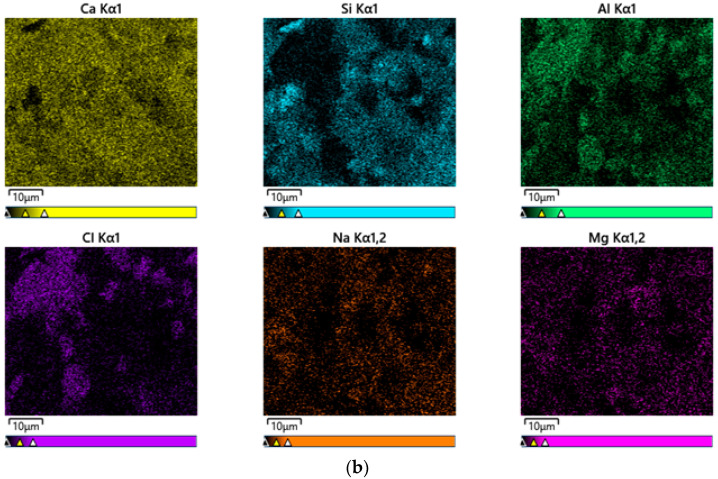
EDS mapping results of 28 d samples. (**a**) CF20 and (**b**) NF20.

Figure 11 shows EDS mapping results for the CF20 and NF20 samples prepared with 20% FSA. Notably, both samples comprise similar elements, such as Ca, Si, Al, Cl, Na, and Mg, regardless of the type of alkaline activator. This result might be because, as described by Li et al. [39], the constituent elements of the alkali-activated composites are more influenced by the type of binder used than by the type of alkaline activator used.

### 3.7. Thermogravimetric Analysis

**Figure 12:**
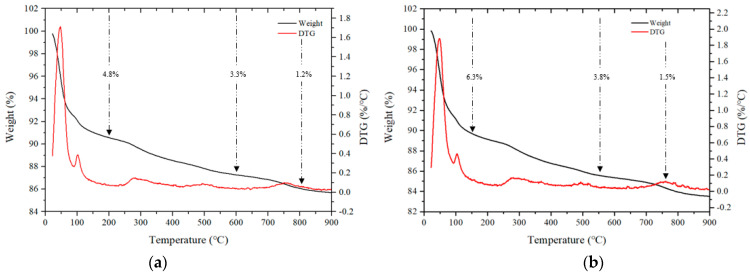

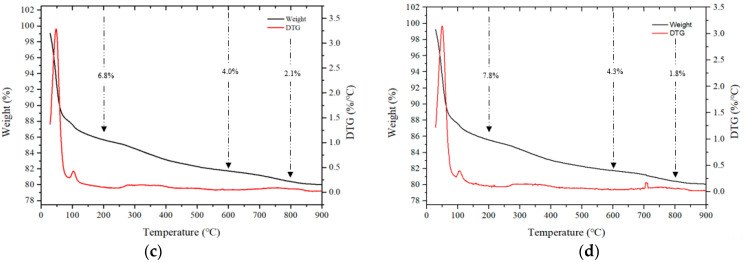
TGA of samples after 28 d. (**a**) CF00, (**b**) CF20, (**c**) NF00, and (**d**) NF20.

Figure 12 shows the TGA results for some samples after 28 d. Mass loss occurring in three stages is generally observed during TGA [40,41]. In the first stage, in a temperature range of 50–200 °C, the bound water and moisture contained in the C–(A)–S–H gel evaporates, and in the second stage, which spans from 200 to 600 °C, mass loss occurs due to thermal decomposition of the hydrate. In addition, the third stage loss that occurs in the range of 600 to 800 °C is caused by the decomposition of calcium carbonate at high temperatures.

For samples using CaO, the loss rates in the range of 50 to 200 °C for the CF00 and CF20 samples were 4.8% and 6.3%, respectively, and the CF20 sample with 20% FSA showed a relatively large loss rate.

Even for samples with Na_2_SiO_3_, loss rates ranging from 50 to 200 °C for the NF00 and NF20 samples were 6.8% and 7.8%, respectively, indicating a high loss rate for samples with FSA. Moreover, in the range from 200 to 600 ℃, the loss rate of samples with FSA was higher than that of the samples without FSA regardless of the type of alkali activator. Therefore, it can be assumed that more hydrates were generated in the alkali-activated sample with FSA, leading to a high 28 d compressive strength [42].

The loss rate in the range of 600 to 800 °C was generally similar. In particular, the loss rate was between 1.2% and 1.5% for CaO samples and between 1.8% and 2.1% for Na_2_SiO_3_ samples.

## 4. Conclusions

1. For the same amount of FSA, the mortar flow of the sample with Na_2_SiO_3_ as the activator was larger than that of the sample with CaO. Additionally, mortar flow increased as FSA content increased regardless of the type of alkaline activator.
2. The maximum temperature of CF10, CF20, and CF30 samples containing FSA was between 35.4 and 36.4 °C, which was between 3.8% and 6.7% higher than that of the CF00 sample. Moreover, the time to reach the maximum temperature for the CaO and Na_2_SiO_3_ samples tended to be slightly delayed as the FSA content increased.
3. The 7 d compressive strength of the sample with CaO was higher than that with Na_2_SiO_3_. However, the 28 d compressive strength of the NF20 sample with Na_2_SiO_3_ and 20% FSA was the highest at approximately 55.0 MPa. In addition, the compressive strength of the sample increased up to a 20% FSA ratio, but when the FSA ratio exceeded 20%, the compressive strength decreased.
4. The tensile strength of the sample with 20% FSA was 3.4 (sample with CaO) and 3.1 MPa (sample with Na_2_SiO_3_), which was approximately 13.3% to 14.8% higher than that of the sample with 0% FSA.
5. After 7 d, the total charge passing through the sample with Na_2_SiO_3_ was approximately 1.79 to 2.24 times higher than that of the sample with CaO. Moreover, the total charge decreased when the amount of FSA increased. In particular, the total charge of the CF30 sample using 30% FSA was approximately 907 C, corresponding to a “very low” value according to ASTM C 1202. After 56 d, the total charge values of all samples decreased significantly.
6. In the range from 200 to 600 °C, the mass loss rate of the samples with the FSA was higher than that of the samples without the FSA regardless of the type of alkali activator. Therefore, more hydrates were generated in the alkali-activated sample with FSA, leading to a high 28 d compressive strength.

The results of this study showed that when FSA was mixed up to 20%, it showed effective results for compressive strength and splitting tensile strength, and when FSA was mixed, it was shown that chloride ion penetration resistance increased. In addition, the performance of the alkali-activated composite containing FSA was superior in the early age when CaO was used, and when Na_2_SiO_3_ was used, the early age reaction was slow, but performance in the long-term age was superior to that of the sample using CaO.

## Figures and Tables

**Table 1:** Chemical composition of cementitious materials.

Type	SiO_2_	Al_2_O_3_	Fe_2_O_3_	CaO	MgO	K_2_O	Blaine (cm^2^/g)	Density (g/cm^3^)
Ground granulated blast-furnace slag (GGBFS)	30.61	13.98	0.32	40.71	6.43	0.60	4210	2.93
Fly ash (FA)	64.88	20.56	6.06	2.58	0.80	1.45	3710	2.21

**Table 2:** Physical properties of fine aggregates.

Type	FM	Density (g/cm^3^)	Water Absorption (%)
Natural sand (NSA)	2.45	2.60	1.0
Ferronickel slag aggregate (FSA)	3.64	3.04	0.6

**Table 3:** Chemical composition of FSA.

	Si	Al	Fe	Ca	Mg	K
FSA	25.1	2.7	9.6	1.9	16.6	0.5

**Table 4:** Mix proportions of alkali-activated composites.

Mix.	W/B (%)	W (kg/m^3^)	NSA (S*%)	FSA (S*%)	GGBFS (kg/m^3^)	FA (kg/m^3^)	CaO (B*%)	Na_2_SiO_3_ (B*%)	Ca(HCOO)_2_ (B*%)	CaCl_2_ (B*%)	NaOH (B*%)
CF00	40	160	100	-	300	40	2.5	-	5	5	2.5
CF10			90	10			2.5	-			
CF20			80	20			2.5	-			
CF30			70	30			2.5	-			
NF00			100	-			-	2.5			
NF10			90	10			-	2.5			
NF20			80	20			-	2.5			
NF30			70	30			-	2.5			

## Data Availability

The original contributions presented in the study are included in the article material, further inquiries can be directed to the corresponding author.
